# Osteoporotic fractures of proximal femur: clinical and epidemiological features in a population of the city of São Paulo

**DOI:** 10.1590/S1516-31802001000200002

**Published:** 2001-03-02

**Authors:** Ana Claudia Ramalho, Marise Lazaretti-Castro, Omar Hauache, José Gilberto Vieira, Edmilson Takata, Francisco Cafalli, Fernando Tavares

**Keywords:** Osteoporotic fracture of proximal femur, Osteoporosis, Physical activity, Dairy calcium ingestion, Body mass index, Fratura proximal de fêmur osteoporóticas, Densidade mineral óssea, Índice de massa corpórea, Ingesta de cálcio, Atividade física

## Abstract

**CONTEXT:**

It is believed that about 25% of menopausal women in the USA will exhibit some kind of fracture as a consequence of osteoporosis. Fractures of the proximal femur are associated with a greater number of deaths and disabilities and higher medical expenses than all the other osteoporotic fractures together.

**OBJECTIVE:**

To study the clinical and epidemiological features of patients with proximal femur fracture in hospitals in São Paulo.

**DESIGN:**

Transversal and retrospective study.

**LOCAL:**

Hospital São Paulo and Hospital Servidor Público Estadual "Francisco Morato Oliveira".

**PARTICIPANTS:**

Patients aged sixty-five years or more hospitalized because of proximal femur fracture, from March to November 1996 (N = 73). This group was compared to patients of the same age without fracture of the proximal femur.

**INTERVENTION:**

Evaluation of weight, height, body mass index; lifestyle habits (physical activity at home, ingestion of dairy calcium, drinking of coffee, smoking habit), gynecological history (ages at menarche and menopause, number of pregnancies and lactations), previous morbidity, use of medications, history of previous fractures, family history of osteoporosis.

**MEASUREMENT:**

The comparison of the different data regarding lifestyle habits between the two groups was made using the chi-squared test. Other data were analyzed using the Mann – Whitney test. P £ 0.05 was considered significant.

**RESULTS:**

We noted a predominance of proximal femur fracture among females in relation to males (a female/male ratio of 3.3:1) with a progressive increase in the frequency of proximal femur fracture with age in both sexes. The group with proximal femur fracture, in comparison with the control group, showed a lower body mass index, less physical activity, and a greater number of pregnancies and lactations. Other data were not different.

**CONCLUSION:**

In accordance with the literature, we found a predomination of proximal femur fracture in women in relation to men, and a favorable effect of higher body mass index and physical activity for decreasing the frequency of proximal femur fracture. We also discuss the role of pregnancies and lactation on the frequency of proximal femur fracture.

## RESUMO

**CONTEXTO:**

Acredita-se que por volta de 25% das mulheres menopausadas nos EUA venham a sofrer de alguma fratura óssea em decorrência da osteoporose. Fraturas proximais de fêmur são associadas com maior número de mortes, incapacidade e custo médico maior do que as outras fraturas osteoporóticas juntas (vertebral e rádio distal).

**OBJETIVO:**

Avaliar características clínicas e epidemiológicas de pacientes com fratura proximal de fêmur em hospitais de São Paulo.

**TIPO DE ESTUDO:**

Transversal retrospectivo.

**LOCAL:**

Hospital São Paulo e Hospital do Servidor Público Estadual "Francisco Morato Oliveira".

**PARTICIPANTES:**

Pacientes com idade igual ou superior à 65 anos, internados com fratura proximal de fêmur nos hospitais acima citados entre março e novembro de 1996 (n = 73). Esse grupo foi comparado a pacientes ambulatoriais, de mesma idade, sem fratura proximal de fêmur (n = 50).

**INTERVENÇÃO:**

Avaliação de dados antropométricos (peso, altura, índice de massa corpórea), hábitos de vida (atividade física, tabagismo, ingesta de cálcio, álcool e café), antecedentes ginecológicos (menarca, menopausa, gestações e amamentações), tipo de fratura, doenças concomitantes e medicações em uso.

**VARIÁVEIS ESTUDADAS:**

Para comparação dos diferentes hábitos entre os dois grupos, utilizamos o teste do Qui-quadrado; demais dados foram comparados por teste não paramétrico de Mann-Whitney; sendo significante P £ 0, 05.

**RESULTADOS:**

Observamos predomínio de fratura proximal de fêmur no sexo feminino (3, 3 mulheres/1 homem) com aumento progressivo da frequência de fratura proximal de fêmur com avançar da idade em ambos os sexos. O grupo com fratura proximal de fêmur comparado ao controle, apresentou menor índice de massa corpórea, menor atividade física, maior número de gestações e amamentações. Demais dados sem diferença estatística.

**CONCLUSÃO:**

Concordante com a literatura, constatamos um predomínio de fratura proximal de fêmur no sexo feminino, o efeito benéfico do índice de massa corpórea mais elevado e da atividade física na redução da freqüência de fratura proximal de fêmur. Salientamos ainda, o papel da gestação e lactação sobre as fraturas proximais de fêmur.

## INTRODUCTION

It is believed that about 25% of menopausal women in the USA will exhibit some kind of fracture as a consequence of osteoporosis. ^1^ Fractures of the proximal femur are associated with a greater number of deaths and disabilities and higher medical expenses than all the other osteoporotic fractures together.^2,3^ The incidence of these fractures has doubled in the last 25 years and it is estimated that 6,000,000 individuals in the world will suffer fracture of the proximal femur in 2050.^4^ As the elderly population continues to increase, this fracture is regarded as an orthopedic epidemic.^2^ This results in cost increases for various countries and it therefore represents a great social and economic problem.

The annual numbers of cases of proximal femur fracture vary widely among different countries and races, and within the same country different races can exhibit different incidences. In Brazil, epidemiological studies of proximal femur fracture are scarce. In the USA white women have twice the risk of fractures in comparison with black women.^5^

In a study of hip fractures in a region of Santiago, Chile, women showed an incidence 1.5 times greater than men and, in comparison with English rates, the Chilean rates are six times lower in women and three times lower in men.^6^ These differences demonstrate the importance of evaluating the epidemiological features of proximal femur fracture in different countries, and specifically in Brazil, where the information in this area is scanty.

The frequency of proximal femur fracture is correlated with various factors such as: osteoporosis, ^7^ decrease of muscle force with age, ^8^ geometry of the hip, ^9^ maternal history of hip fracture and/or personal history since menopause, ^7^ ingestion of calcium and vitamin D, ^10^ factors that influence falls^8^ and genetic predictors.^11^

Our objective was to study the clinical and epidemiological features of patients with proximal femur fracture in hospitals in São Paulo.

## METHODS

The procedures that follow were in accordance with the ethical standards of the committee responsible for human experimentation and with the Helsinki Declaration of 1975, as revised in 1983.

We studied 73 patients admitted into the orthopedic service of the Federal University of São Paulo/Escola Paulista de Medicina and Hospital do Servidor Público Estadual "Francisco Morato Oliveira", São Paulo, due to proximal femur fracture, aged 65 years or more. Patients with fractures of tumoral or metastatic etiology were excluded. The patients studied were compared with a group of patients of the same age and without proximal femur fracture (n = 50), seen in the geriatric outpatients service of the Federal University of São Paulo/Escola Paulista de Medicina.

Both groups underwent an evaluation of weight, height and body mass index. They also answered a questionnaire to evaluate age, physical activity at home, ingestion of dairy calcium, drinking of coffee, smoking habit, ages at menarche and menopause, number of pregnancies and lactations, previous morbidity, use of medications, history of previous fractures, cause of current fracture. The fracture type (transtrochanteric or neck of femur) and fracture management (surgical or clinical) was checked in the record.

Weight and height were used to calculate the body mass index and were supplied by the patients if they were in bed because of locomotion inability. The body mass index is an index described last century by a Belgian astronomer and is calculated using weight and height [body mass index = weight in kilograms/(height in meters)^2^].

Physical activity at home was evaluated in three classes: 1- those who did not perform any activity; 2- those who had an activity of less than 4 hours a day; 3- those who had an activity of more than 4 hours a day. Among these activities the following acts were considered: walking in and out of the house, going upstairs and downstairs, time spent in washing clothes, taking care of the kitchen, arrangement, tidying up and upkeep of the house and garden.

Ingestion of dairy calcium was characterized by the quantity of glasses of milk drunk in a day, as well as the ingestion of milk byproducts (yogurts, cheeses, etc.). From these data the patients were classified into three groups: 1- those with calcium ingestion less than 500 mg/ day; 2- between 500 and 1000 mg/day and 3- greater than 1000 mg/day.

Drinking of coffee was highlighted when it was more than 6 demitasses a day. A history of alcoholism was recorded when 3 measures/ day had been taken for more than 10 years. Smoking was considered when its use was greater than 10 cigarettes a day for more than 10 years.

The cause of fractures was characterized as being due to falls or spontaneous. They were considered as spontaneous when only a pain upon movement was described, with the absence of a fall. In the cases of falls the sites of the falls were investigated.

Previous morbidity and medications that were mentioned by the patients were checked in the admission record. In the group with proximal femur fracture there were 23.2% with arterial hypertension, 12.3% with cardiopathy, 8.2% with senile dementia, 6.8% with arthrosis and 1.4% with hypothyroidism. The use of thiazides was recorded in 15%. Other medications used were oral hypoglycemic agents in 12.3%, anti-hypertensive agents in 17.8% and anxiolytic and/or psychotropic agents in 4.1%.

In the group of patients without proximal femur fracture there were 14% with diabetes, 54% with arterial hypertension, 4% with cardiopathy, 10% with dyslipemia, 6% with bronchitis, 6% with arthrosis and 2% with hypothy roidism. Patients with neoplasia were excluded. The use of thiazides was recorded in 18%. Other medications used were oral hypoglycemic agents in 8%, anti-hypertensive agents in 28%, anxiolytic and psychotropic agents in 2%.

The same interviewer filled out the all the questionnaires. The protocol had been submitted to and approved by the committee for medical ethics of the Escola Paulista de Medicina and Hospital do Servidor Público Estadual "Francisco Morato Oliveira", São Paulo.

### Statistical Analysis

The data were presented as average, standard deviation (SD) and median, but analyzed using a non-parametric test (Mann-Whitney), since most of the data with the exception of body mass index did not exhibit a normal distribution. The comparisons between the clinical data like calcium ingestion, activity at home, smoking, alcoholism, coffee drinking, number of pregnancies and breast-fed children, and type of fracture was made using c^2^ (Chi-square test).

The statistical analyses were made using the Sigma-Stat Program (Jandel Corporation, USA). P £ 0.05 was considered significant.

## RESULTS

**Figure 1:**
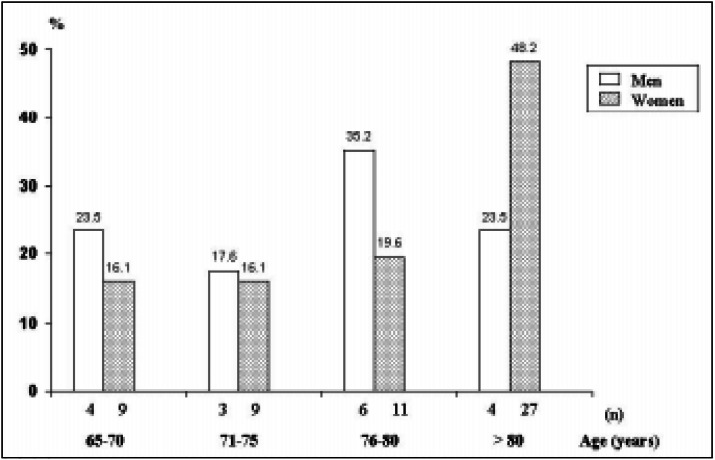
Age and sex distribution of patients with proximal femur fracture.

In the group of elderly patients with proximal femur fracture, we noted a predominance of female over males, in a ratio of 3.3:1. The average age was 78.5 (SD 7.2) years and the median was 78 years (range of 65 to 94 years). The group of men had an average age of 76.5 (SD 6.4) years with a median of 76 years, while for the women it was 79.1 (SD 7.4) with a median of 80 years (P = 0.18). By dividing them into subgroups according to different age levels, a geometric increase in the frequency of proximal femur fracture was observed above the age of 70 years, with 42.4% of the cases occurring in elderly patients over 80 years of age. The greatest number of cases of proximal femur fracture in men occurred from the age of 75 years, when the number of proximal femur fractures is doubled (58.7%), while for women there was an almost doubling of frequency later, starting at the age of 80 years (48.2%) (Figure 1).

As for the racial distribution in both groups there was, a predominance of whites over the others (76.7% in the group with proximal femur fracture and 76% in the group without proximal femur fracture). The number of blacks was small in both groups with 1.4% in the group with proximal femur fracture and 10% in the group without proximal femur fracture. The number of mixed-race was the same in the two groups: 16.4% in the group with proximal femur fracture and 14% in the group without proximal femur fracture.

In the groups of patients with proximal femur fracture and patients without proximal femur fracture, various aspects were compared in an attempt to distinguish factors that could be associated with the occurrence of proximal femur fracture. The features of these two groups are shown in Table 1.

**Table 1:** Different epidemiological characteristics of the studied groups: the group with proximal femur fracture and the group without proximal femur fracture. SD = standard deviation, BMI = Body Mass Index, n = number, P ≤ 0,05 was considered significant

	group with proximal femur fracture		group without proximal femur fracture		
Variables	Mean (SD)	Median	Mean (SD)	Median	p
Age (years)	78.5 (7.1)	78	72.9 (5.2)	72	< 0.001
Weight (kg)	61.8 (13.2)	60	65.5 (10.4)	66	0.13
Height (m)	1.6 (0.1)	1.6	1.6 (0.0)	1.6	0.659
BMI [weight/(height)^2^]	24.5 (4.2)	24.6	26.3 (4.5)	25.8	0.02
Age of menarche (years]	13.5(1.6)	13	13 (1.7)	13	0.276
Age of menopause (years]	49.3 (5.0)	50	48.4 (3.6)	49.5	0.355
N° of Pregnancy	3.2 (2.7)	3	2.2 (2.6)	2	0.0267
N° of children breasted-feed	2.8 (2.6)	3	1.6 (1.7)	1	0.0143

Of the studied features, age, body mass index, physical activity, number of pregnancies and lactations were statistically different between the two groups.

body mass index than did the elderly without proximal femur fracture.

Most of the women in both groups were multiparous. The group with proximal femur fracture presented 14.3% nulliparous, while in the group without proximal femur fracture there were 25% (P = 0.3). The number of pregnancies and lactations was significantly greater in women with proximal femur fracture than in women without proximal femur fracture (Table 1).

The average age at menopause and menarche in both groups was not different. Eight (14.2%) women with proximal femur fracture and 5 (13.9%) without proximal femur fracture did not remember the age of menarche while 13 (23.3%) of the women with proximal femur fracture and 15 (41.6%) without proximal femur fracture did not remember the age of menopause.

Comparatively, the two groups did not differ as to ingestion of calcium, as shown in Table 2. In both groups, most of the individuals (68.5% of the elderly with proximal femur fracture and 80% without proximal femur fracture) ingested ≤ 500 mg/day.

Comparing the two groups, no statistically significant difference was observed as to coffee drinking, alcoholism and smoking.

Of the patients with proximal femur fracture, 8 (11%) had a history of more than 6 demitasses of coffee compared with 12 (24%) of the patients without proximal femur fracture (P = 0.094). A history of chronic alcoholism was noted in 5 (6.8%) of the group with proximal femur fracture and 6 (12%) of the group without proximal femur fracture (P = 0.508). Smoking was recorded in 15 (20.5%) of the group with proximal femur fracture and 15 (30%) in the group without proximal femur fracture (P = 0.324).

**Figure 2:**
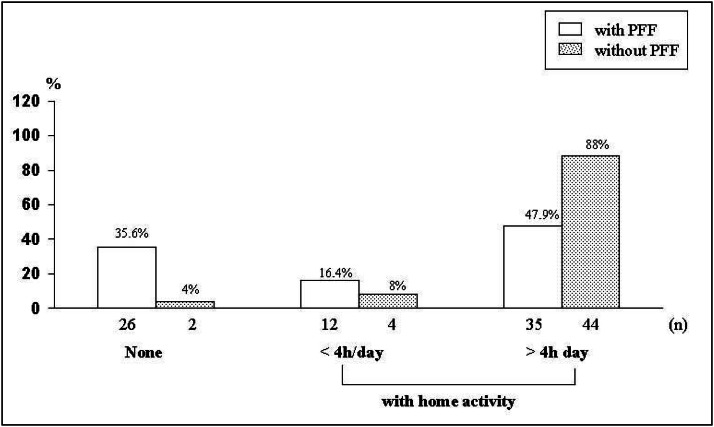
Distribution by frequency of patients with proximal femur fracture (PFF) and controls, according to the duration of physical activity during one day, like walking, working at home, in the garden or in the kitchen, keeping the house etc. The horizontal axis represents the various levels of physical activity depending on the duration (none = elderly individual without any activity; < 4 h/day = elderly individual with up to 4 hours a day of physical activity; > 4 h/day = elderly individual with more than 4 hours a day of physical activity.

Regarding activity at home, there was a significant difference between the groups with and without proximal femur fracture. The group without proximal femur fracture was much more active and 88% mentioned activities at home lasting ≥ 4 hours/day compared to 47.9% of the patients with proximal femur fracture (χ^2^= 22 and P < 0.0001) (Figure 2).

The history of previous fractures in the 2 groups was positive in 28 (38.4%) and 13 (26%), respectively, for patients with and without proximal femur fracture (P = 0.22). Of these fractures the most frequent was wrist fracture in both groups: 35.7% of the 28 cases in the group with proximal femur fracture and 46.7% of the 13 cases without proximal femur fracture. Eight patients had already had a fracture in the opposite femur.

Evaluating the type of present fracture of the proximal femur, 37 (50.7%) cases of fracture of the femur neck and 36 (49.3%) cases of trochanteric fractures were observed. However, when we reevaluated the type of fracture in men and women, there was a predominance of the transtrochanteric fractures in men (58.8%) in comparison with women (46.4%), although without statistical significance. Analyzing the types of fractures in relation to the age groups, we noticed that the average age for the transtrochanteric fractures was 79.6 (SD 7.1) years, with a median of 80 years, while for fractures of the femur neck it was 77.4 (SD 7.3) years with a median of 77 years (P = 0.2). There was a greater concentration of patients with transtrochanteric fractures over 80 years of age (47.2%) and few between 65 to 70 years (11.1%), when compared with patients that presented femur neck fractures, of whom 37.8% were aged over 80 years and 24.3% between 65 and 70 years. This difference was, however, not statistically significant (P = 0.5).

Investigating the cause of the fracture, we recorded a history of falls in 64 (87.7%) cases and in 9 (12.3%) falls were not mentioned. Of these 64 cases of falls, 58 (90.6%) occurred at home. In the cases without a history of falls, there was spontaneous pain provoked by motion such as rising from a chair or from a bed.

In the vast majority of the cases, the management was surgical (93.2%) and clinical follow-up was chosen in only 5 (6.8%) cases.

## DISCUSSION

Epidemiological studies on proximal femur fractures are scant in Brazil. This work was the first to do an epidemiological characterization in relation to various aspects of a population in São Paulo. The epidemiological characterization of such a group with proximal femur fracture in different geographic areas has crucial importance because there are differences that depend on the ethnic ancestry, genetic and environmental factors of each population.

Our group with proximal femur fracture showed a female/male ratio 3.3:1. This ratio between the sexes is very similar to the ratio found in the region of La Plata, Argentina (3.8:1), ^12^ or in Oxford, England (3:1).^13^ In Rome, Italy, the ratio was 4.5:1.^14^

The median age of 80 years for women was similar to that one found in Argentina.^12^ Men were, on average, younger with a median of 76 years, a result that is also similar to the Argentine data, in which the median was 77 years.^12^ As described before, ^2,6,12,15^ there is an exponential growth in proximal femur fractures with advancing age.

Farmer et al.^5^ suggested that the risk of proximal femur fracture doubles every 5 years after 50 years of age. In our study we have not observed variations in the percentage of cases between 65 to 70 years and 71 to 75 years. However, from half-way through the eighth decade there was a significant exponential increase in frequency of proximal femur fracture, with the highest risk being in patients over 80 years (42.4%). One interesting feature seen in this study was that the frequency of cases of proximal femur fracture doubled among men from the age of 75 years, while for women this occurred at over 80 years of age. We noted that even after the age of 75 years, women have a risk of proximal femur fracture almost four times higher than for men (3.8:1). This sex-dependent difference in osteoporotic fractures is partly explained by the lower bone mass peak among women^16^ and the rapid bone loss associated with the menopause.^16^

The risk of proximal femur fracture varies widely according to racial groups. Farmer et al.^5^ in the USA found a risk of proximal femur fracture twice as large among white women as among black women. In our study we noted a low frequency of blacks in the group with proximal femur fracture (1.4%), although this can not really evaluate the risk ratio between blacks and whites. To do that it would be necessary to increase the number of participants in the study and to study populations from other districts of São Paulo.

In our study of the clinical features of the groups of elderly patients with and without proximal femur fracture, we observed a significant difference in body mass index between the groups. The group with proximal femur fracture presented a lower average body mass index. This finding has been recorded by other authors.^17,18^ There are different possibilities for explaining why elderly individuals with higher body mass index suffer fewer fractures. Firstly, both muscle and fat tissue increase the total body weight and augment the weight stress on the bone, avoiding bone loss.^17^ Secondly, fat is a source of estrone in menopausal women, by the conversion of androstenedione to estrone, and estrogen has an anti-resorptive effect on the bone.^17^ And lastly, fat can function as a cushion for the hip, softening the falls.

The number of pregnancies was on average greater in women with proximal femur fracture than in those without proximal femur fracture. Despite the clear effects of pregnancy on calcium metabolism, its effect on bone mineral density and risk of fracture is still not clear. Both positive and negative associations between the number of pregnancies and the bone mineral density have been reported. Drinkwater et al.^19^ reported in a prospective study a reduction in bone mineral density in the femur neck during pregnancy, which continued to decrease during the 6 months of lactation. Hreshchyshyn et al.^20^ reported a similar finding and predicted a reduction of 1.1% in bone mineral density of the femur neck during each pregnancy. With opposite results, Christiansen et al., ^21^ Goldsmith et al.^22^ and Alderman et al.^23^ did not record bone loss during pregnancy.

There is little data in the literature about the effect of pregnancies on menopausal fractures, and not only on body mass index. In contradiction to our results, Alderman et al.^23^ did not find a significant difference in the number of pregnancies between women with wrist fractures or proximal femur fractures and the controls. There is little previous data in the literature on increases in the risk of osteoporotic fractures of the proximal femur related to parity.

In our study we found such an association. Probably the cumulative loss of bone mineral density in the femur neck during several pregnancies in multiparous women (as demonstrated in the above studies) superimposed on a lower initial bone mass can explain why the number of pregnancies was greater in women with proximal femur fracture in relation to women without proximal femur fracture. In addition to this, it is important to consider the socioeconomic and cultural factors of the population we examined, which, unable to provide the physiological calcium needs during pregnancy (>1200 mg/day), may have its bone mineral density compromised at each pregnancy and this in the postmenopausal period, with the addition of other factors, increases the risk of proximal femur fracture.

In relation to lactation, we observed that women with proximal femur fracture, on average, suckled more than did women without proximal femur fracture. This can be explained by the decrease in bone mineral density during lactation. This reduction during lactation is seen in most of the reports that have studied this subject.^19,22,24,25^ Sowers et al.^26^ verified a reduction in bone mineral density among women that suckled for more than 6 months, with the return to the basal level of bone mineral density occurring 12 months after childbirth. The reduction in bone mineral density during lactation is explained by the decrease in estrogen levels due to the decrease in follicle-stimulating hormone (FSH), caused by the increase of prolactin.^26^

On the whole, evaluation of the controversial studies on the effects of parity and lactation on bone mineral density and later risk of osteoporotic fractures is very difficult because there are some factors that are potentially capable of hampering comparative evaluation of the results. Among these factors, the following can be cited: weight gain during pregnancy or weight change after parturition, history of abortion or fetal loss, interval between lactation and next pregnancy and age at first pregnancy. This latter item can be of crucial importance because a first pregnancy at an earlier age can impair the bone mass peak.^27^ Thus, the affirmation that women's numbers of pregnancies and lactations influence their bone mineral density and their risk of postmenopausal fractures is true when based on the evaluation of the factors discussed above.

As also reported in other studies, ^18,28-30^ we did not find an association between dairy calcium intake and protection from proximal femur fracture, even in women with very low intake. Holbrook et al.^31^ evaluated calcium ingestion with a complete food questionnaire and found a relationship between low calcium ingestion and an increased risk of fractures.

As also seen in other studies, ^17,28-30^ we found a retrospective history of less activity at home

**Table 2:** Ingestion of dairy calcium in the groups with and without proximal femur fracture (PFF) during one day

	Ingest of dairy calcium (mg/day)		
Groups	< 500	500-1000	> 1000
With PFF	25 (34.3%)	25 (34.2%)	23 (31.5%)
Without PFF	20 (40%)	20 (40%)	10 (20%)

*χ^2^ = 2; P = 0,36.*

As also seen in other studies, ^17,28-30^ we found a retrospective history of less activity at home in the group with proximal femur fracture in comparison with the group without proximal femur fracture. Cooper et al.^29^ also verified that in both sexes an increase in daily activity, including walking, going upstairs, housework and gardening, protects against proximal femur fracture. The more active elderly individuals exhibit greater muscular force, which favors a greater charge on the bone, improving bone mineral density.^17^

Contrary to some authors^18,30,32^ we did not find an association between coffee drinking, smoking or the use of alcohol and proximal femur fracture.

The history of previous fractures was not different between the two groups. Bagur et al., ^12^ evaluating proximal femur fracture in Argentina, found that 8% of the patients exhibited a previous fracture in the contralateral femur, a finding that was similar to ours (10%). In their study, 15% of the patients with proximal femur fracture reported a history of wrist fracture, a figure that is very close to ours (12%). However, in our study we cannot affirm that the presence of wrist or other types of fracture can predict the risk of proximal femur fracture, as a similar number of fractures at other sites were referred to in the group without proximal femur fracture.

Regarding the type of fracture, Bagur et al.^12^ in Argentina found a predominance of transtrochanteric fractures (58%) over those of the femur neck (39%) among men. In our study, we found a tendency towards a greater number of transtrochanteric fractures in men (58.8%) in relation to those of the femur neck (41.1%), but without statistical significance (P = 0.53). The average age for these two types of fracture exhibited a tendency to be higher in the group with transtrochanteric fractures in relation to the fractures of the femur neck, but without statistical significance (P = 0.2). In the elderly patients over 80 years old, 41.9% of fractures were of the femur neck and 58.1% transtrochanteric. On the other hand, in the elderly from 65 to 70 years old the percentage of femur neck fractures was 69.2% and for transtrochanteric it was 30.7%. This confirms the observed tendency that with advancing age, elderly patients exhibit an increase in the proportion of transtrochanteric fractures in relation to the femur neck.

According to the literature, ^8,16,35^ the cause of fractures has been related to falls in 86.3% of the cases, and 92.1% of these falls occurred at home. Dargent-Molina et al.^8^ emphasized the importance of risk factors for falls in women above 75 years of age as the determining factor for proximal femur fracture, as well as for the reduction in bone mineral density. This finding emphasizes the importance of interventions aimed at decreasing the risk factor for falls, like trying to reduce the use of drugs that adversely influence balance, the use of methods for improving the support when walking, climbing stairs, and the use of appropriate shoes.

Proximal femur fracture constitutes a serious socioeconomic problem because of its high morbidity and mortality. Such fractures are presented with features that vary among different populations with particular racial and environmental factors, and thus studies like this take on fundamental importance. As such studies determine the predisposing factors, prophylactic therapy can be applied to individuals characterized as being at risk of proximal femur fracture. In this studied population, we can conclude that an increase in body mass index, physical activity and dairy calcium ingestion may constitute prophylactic measures against proximal femur fractures.

Our study showed a higher frequency of proximal femur fractures among females in relation to males and a progressive increase in the frequency of proximal femur fractures with age. We noted a favorable effect of a higher body mass index and physical activity on protection against proximal femur fractures. We also noted a negative effect of the number of pregnancies and lactations on the frequency of proximal femur fracture. To better evaluate the frequency of proximal femur fracture in relation to race, we need a greater number of individuals in the study and also an evaluation of people from different districts of São Paulo.
